# Comparison on Response and Dissolution Rates Between Ursodeoxycholic Acid Alone or in Combination With Chenodeoxycholic Acid for Gallstone Dissolution According to Stone Density on CT Scan: Strobe Compliant Observation Study

**DOI:** 10.1097/MD.0000000000002037

**Published:** 2015-12-18

**Authors:** Jae Min Lee, Jong Jin Hyun, In Young Choi, Suk Keu Yeom, Seung Young Kim, Sung Woo Jung, Young Kul Jung, Ja Seol Koo, Hyung Joon Yim, Hong Sik Lee, Sang Woo Lee, Chang Duck Kim

**Affiliations:** From the Department of Internal Medicine (JML, JJH, SYK, SWJ, YKJ, JSK, HJY, HSL, SWL, CDK); and Department of Radiology (IYC, SKY), Korea University College of Medicine, Seoul, Korea.

## Abstract

Medical dissolution of gallstone is usually performed on radiolucent gallstones in a functioning gallbladder. However, absence of visible gallstone on plain abdominal x-ray does not always preclude calcification. This study aims to compare the response and dissolution rates between ursodeoxycholic acid (UDCA) alone or in combination with chenodeoxycholic acid (CDCA) according to stone density on computed tomography (CT) scan.

A total of 126 patients underwent dissolution therapy with either UDCA alone or combination of CDCA and UDCA (CNU) from December 2010 to March 2014 at Korea University Ansan Hospital. In the end, 81 patients (CNU group = 44, UDCA group = 37) completed dissolution therapy for 6 months. Dissolution rate (percentage reduction in the gallstone volume) and response to therapy (complete dissolution or partial dissolution defined as reduction in stone volume of >50%) were compared between the 2 groups. Dissolution and response rates of sludge was also compared between the 2 groups.

The overall response rate was 50.6% (CNU group 43.2% vs UDCA group 59.5%, *P* = 0.14), and the overall dissolution rate was 48.34% (CNU group 41.5% vs UDCA group 56.5%, *P* = 0.13). When analyzed according to stone density, response rate was 33.3%, 87.1%, 30.0%, and 6.2% for hypodense, isodense, hyperdense, and calcified stones, respectively. Response rate (85.7% vs 88.2%, *P* = 0.83) and dissolution rate (81.01% vs 85.38%, *P* = 0.17) of isodense stones were similar between CNU and UDCA group. When only sludge was considered, the overall response rate was 87.5% (CNU group 71.4% vs UDCA group 94.1%, *P* = 0.19), and the overall dissolution rate was 85.42% (CNU group 67.9% vs UDCA group 92.7%, *P* = 0.23).

Patients with isodense gallstones and sludge showed much better response to dissolution therapy with CNU and UDCA showing comparable efficacy. Therefore, CT scan should be performed before medication therapy if stone dissolution is intended.

## INTRODUCTION

Gallstone is a common cause of abdominal pain, and its prevalence ranges from 5% to 15% among general population.^1,2^ Approximately 1% to 2% of patients become symptomatic every year, and biliary complications occur in approximately 3% of patients after 10 years.^3,4^ Once the patient becomes symptomatic, recurrent biliary pain develops in 38% to 50% of cases.^5,6^ Therefore, cholecystectomy is the standard treatment for symptomatic gallstone disease. However, cholecystectomy cannot always be performed because of severe comorbidity or at times because of patient refusal. In these patients, oral litholysis could be considered if they meet the currently accepted standard criteria for gallstone dissolution: gallstones ≤15 mm in diameter, patent cystic duct, radiolucent on plain abdominal x-ray, functioning gallbladder (GB), and history of biliary pain (right upper quadrant pain ≤30 minutes).^7^ In addition, asymptomatic and mildly symptomatic patients could also be considered potential candidates for gallstone dissolution therapy considering the long life expectancy and increased gallstone incidence among young people, which increases the likelihood of developing complication during their lifetime.^8^

Dissolution therapy of GB stones has been performed since the 1970s with the introduction of chenodeoxycholic acid (CDCA) and ursodeoxycholic acid (UDCA).^9–11^ Although many agents have been introduced for oral litholysis ever since, the most widely used dissolution agents still remain to be UDCA alone or in combination with CDCA. In a meta-analysis on dissolution therapy, the average dissolution efficacy was 33% to 42% and 51% to 74% at best.^12^ There are many factors that affect the efficacy of oral litholysis such as size of the stone, calcification, GB function, and so on. Among these factors, one of the most important factors is the presence of calcification. However, aforementioned currently accepted standard criteria for gallstone dissolution based on the absence of calcification on plain abdominal x-ray seem to be insufficient. This is due to the fact that no < 50% of gallstones which are radiolucent on plain abdominal x-ray appear to be hyperdense on abdominal computed tomography (CT) scan.^13,14^ Another factor is the lack of ability to select cholesterol stones, which are the only stones well known to be responsive to oral litholysis. Up to 20% of pigment gallstones were shown to be radiolucent on plain abdominal x-ray.^15,16^

Previously studies have shown that CT scan was useful in predicting gallstone solubility.^17^ Although the Hounsfield units (HU) of gallstone can provide practical information about stone composition and dissolvability, measuring HU may not always be possible or often unavailable and could also be cumbersome in primary care setting. Using a more intuitive method for categorizing gallstone densities into 4 categories, that is hypodense, isodense, hyperdense, and calcified, could prove to be more practical to primary care physicians. Therefore, we carried out this study to see whether a simplified categorization of gallstone density on CT scan could be useful in selecting patients for gallstone dissolution. We also compared the response and dissolution rates of UDCA alone or in combination with CDCA according to stone density on abdominal CT scan in Korean patients, as the data on the dissolution efficacy of aforementioned 2 most commonly used oral litholytic agents are lacking with the Eastern population.

## MATERIALS AND METHODS

### Patients

A total of 393 patients presented to the outpatient department of Korea University Ansan Hospital with GB stones from December 2010 to March 2014. Among these patients, 126 underwent dissolution therapy with either UDCA alone (n = 64) or in combination with CDCA (n = 62). For combination therapy, magnesium trihydrate of CDCA and UDCA (CNU; Myungmoon Pharm. Co., Seoul, Korea) was used. Each capsule of CNU consists of 114 mg of CDCA and 114 mg of UDCA. Inclusion criteria were gallstones ≤15 mm in diameter, gallstones that were radiolucent on plain abdominal x-ray, and asymptomatic or mildly symptomatic patients. Of these 126 patients, 45 were excluded for the following reasons: follow-up loss (n = 35), symptom development necessitating cholecystectomy (n = 7), and noncompliance (n = 3) which was defined as ingestion of <80% of the prescribed dose. In the end, 81 patients (CNU group = 44, UDCA group = 37) completed an average of 6 months of dissolution therapy and were included for analysis.

### Study Design

**FIGURE 1:**
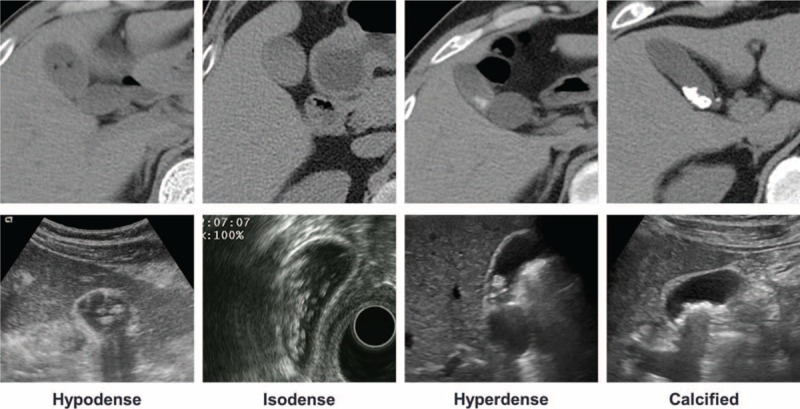
Classification of stone density on CT scan. Images in the above row are representative images of hypodense, isodense, hyperdense, and calcified stones seen on CT scan and those below are corresponding images observed either on abdominal ultrasonography or endoscopic ultrasonography. CT = computed tomography.

Gallstone diameter was measured by abdominal ultrasonography. Change in gallstone diameter was calculated by measuring the largest gallstone in the GB. Gallstone volume was measured using the following equation as for a sphere: 4/3π×r^3^. Dissolution rate was defined as percentage reduction in the gallstone volume. As for the sludge, percentage reduction in number was used as an estimate to assess dissolution rate. Complete dissolution was defined as absence of gallstone on follow-up abdominal ultrasonography. Partial dissolution was defined as reduction in gallstone volume of >50%. Response to therapy was defined as complete dissolution or partial dissolution. Stone density on abdominal CT scan was divided into 4 categories: hypodense, isodense, hyperdense, and calcified (Figure 1). This study was approved by the Institutional Review Board of Korea University Ansan Hospital (AS15140).

### Treatment and Assessment

The treatment consisted of either CNU (3 capsules per day) or UDCA (600 mg per day) administered as 3 divided dose to be taken with the meal. However, if the patients missed taking the medication, they were told to take the missing dose with the next dose. Medications were first prescribed for 2 weeks to assess the occurrence of any side effects. The initially prescribed medication was continued if the patients were tolerable. However, if they were intolerable or complained of any noteworthy side effects, such as diarrhea or abdominal pain, prescription was switched to the corresponding medication, that is from CNU to UDCA and vice versa. Afterward, patients were followed up at 3-month interval. Laboratory tests including complete blood count, blood urea nitrogen, serum creatinine, liver function tests, serum amylase, serum lipase, prothrombin time, and lipid profile were performed at the beginning of treatment, and followed up at 3 months and at 6 months. Abdominal ultrasonography was performed before dissolution therapy, and followed up at 6 months to evaluate for gallstone number and largest diameter of the gallstone. If the patients developed any gallstone-related complications, that is pancreatitis, cholangitis, or cholecystitis, medication was stopped and relevant treatment was given: referral to surgeon for cholecystectomy or endoscopic retrograde cholangiopancreatography for stone removal.

### Statistical Analysis

Statistical analyses were performed using IBM SPSS Statistics version 20.0 (IBM, Armonk, NY). Data are expressed as mean ± SD or n (%) values. Continuous and categorical variables were compared using the Mann–Whitney *U* test and χ^2^ test, respectively. A 2-sided *P* values <0.05 were considered statistically significant.

## RESULTS

### Baseline Characteristics

There were 46 (56.8%) females, and the number of female did not differ between CNU group and UDCA group (*P* = 0.99). Age (49.0 ± 15.0 years vs 52.9 ± 17.2 years, *P* = 0.28), treatment duration (182.4 ± 14.7 days vs 181.5 ± 14.8 days, *P* = 0.79), pretreatment stone size (8.2 ± 3.4 mm vs 9.4 ± 4.7 mm, *P* = 0.27), and stone number distribution (*P* = 0.73) also were not different between the 2 groups. Number of patients with bicameral GB, GB wall thickening, fatty liver, diabetes mellitus, and hypertension were also similar between the 2 groups (Table 1).

**TABLE 1:**
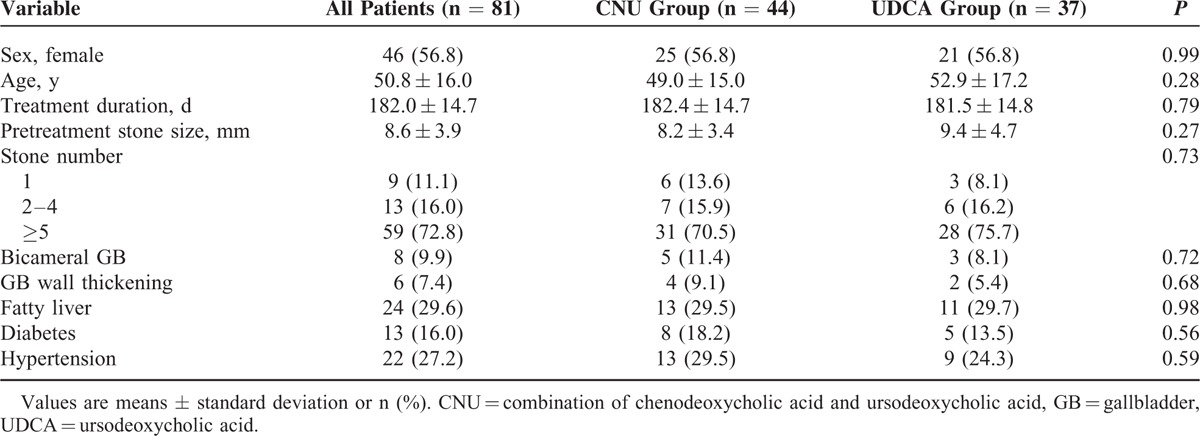
Baseline Characteristics of Patients Undergoing Dissolution Therapy

### Response and Dissolution Rate of CNU and UCDA

The overall response rate of dissolution therapy was 50.6% (41/81). Complete dissolution was achieved in 29.6% (24/81) of patients, and partial dissolution was achieved in 21.0% (17/81) of patients (Table 2). When analyzed according to the medication used for dissolution, the overall response rate was 43.2% (19/44), complete dissolution rate was 18.2% (8/44), and partial dissolution rate was 25.0% (11/44) in the CNU group. As for the UDCA group, the overall response rate was 59.5% (22/37), complete dissolution rate was 43.2% (16/37), and partial dissolution rate was 16.2% (6/37) (Table 2). The overall dissolution rate, measured by percentage decrease in stone volume, was 48.34%; dissolution rate of CNU group and UDCA group was 41.50% and 56.46%, respectively (*P* = 0.13).

**TABLE 2:**
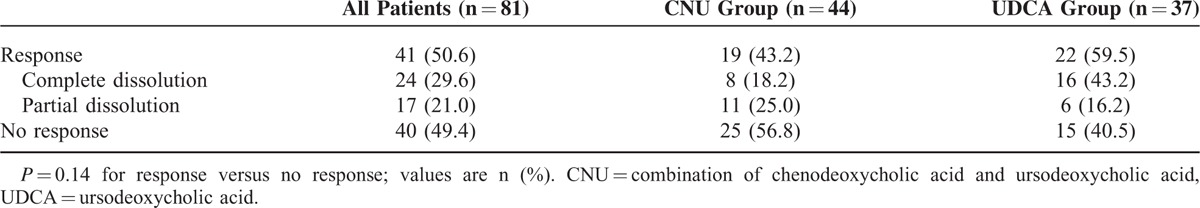
Comparison on Response to Therapy Between CNU and UDCA

### Response and Dissolution Rate According to Stone Density

Among a total of 81 patients who completed dissolution therapy, 70 patients underwent abdominal CT scan before treatment: 36 (81.8%) in the CNU group and 34 (91.9%) in the UDCA group. When response rate was analyzed according to the stone density, response rate was highest with isodense stones (87.1%), followed by hypodense stones (33.3%), hyperdense stones (30.0%), and calcified stones (6.2%) (Table 3). When only isodense stones were selected and compared between CNU group and UDCA group, the response rate was similar (85.7% vs 88.2%, *P* = 0.83) (Table 4). Dissolution rate in this subgroup of patients was 81.01% for CNU group and 85.38% for UDCA group (*P* = 0.17). The overall dissolution rate for isodense gallstone was 83.41%.

**TABLE 3:**
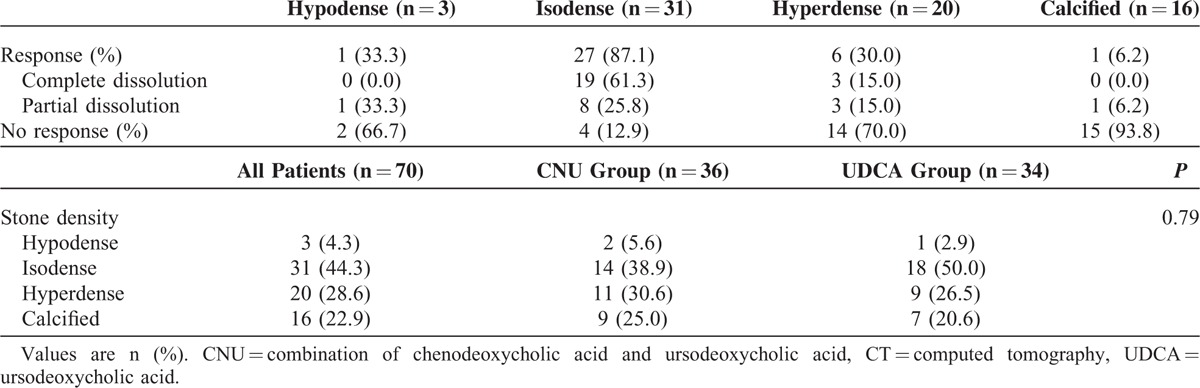
Response to Dissolution Therapy According to Stone Density on Abdominal CT Scan

**TABLE 4:**
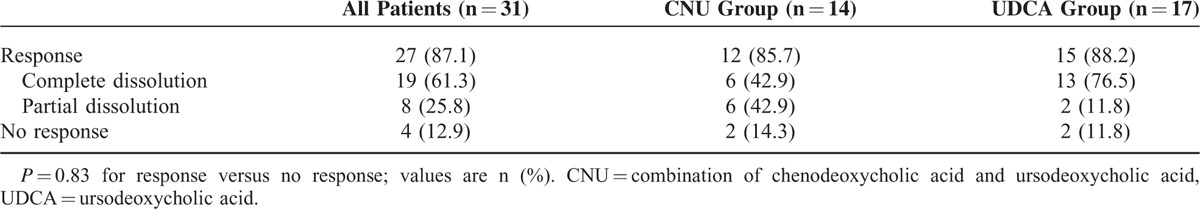
Comparison on Response to Dissolution Therapy of Isodense Gallstones

### Response and Dissolution Rate of GB Sludge

When only 24 patients with sludge were considered, the overall response rate was 87.5%. Although the response rate of UDCA group (94.1%) seemed to be higher than that of CNU group (71.4%), it was not significant (*P* = 0.19) (Table 5). The overall dissolution rate in this subgroup of patients was 85.42%. Dissolution rate was also higher in the UDCA group (92.65%) compared with the CNU group (67.86%), but was not statistically significant (*P* = 0.23). Of the 24 patients with sludge, 22 patients underwent abdominal CT scan before dissolution therapy; 15 (68.2%) had isodense sludge, 5 (22.7%) had hyperdense sludge, and 2 (9.1%) had calcified sludge. Whereas all patients with isodense sludge showed response, the response rate was 80.0% (4/5) for patient with hyperdense sludge and 50.0% (1/2) for those with calcified sludge.

**TABLE 5:**
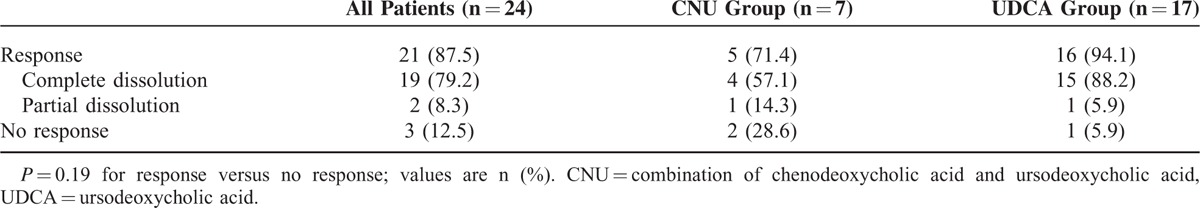
Comparison on Response to Dissolution Therapy of Sludge

## DISCUSSION

The result of our study shows that stone density on abdominal CT scan greatly influences both the response rate and dissolution rate of gallstone dissolution therapy. The response and dissolution rates were greatest in those with isodense stones on CT scan. This clearly demonstrates the benefit of performing CT scan before dissolution therapy to exclude gallstone calcification which cannot be adequately excluded with plain abdominal x-ray. In fact, previous studies have demonstrated that no < 50% of gallstones that were radiolucent on plain abdominal x-ray turned out to be hyperdense on abdominal CT scan.^13,14^ Therefore, it would be recommendable to perform abdominal CT scan to better detect the presence of calcification or calcium content if gallstone dissolution is intended. Nevertheless, one should keep in mind that isodensity on CT scan does not always imply the presence of cholesterol stone because some pigment stones can also appear isodense in the absence of calcium content. As for hypodense stones, the majority of which are considered to be cholesterol stones,^18^ high response and dissolution rates were expected. However, of the 3 patients with hypodense stones, only 1 showed partial response and the remaining 2 showed no response at all. The main reason for this seems to be that those who showed no response had bicameral GB (or segmental adenomyomatosis). Bicameral GB has been shown to be related to the presence of GB stone formation.^19^ It is still unclear whether bicameral GB is the result of previous inflammation or an anomaly in itself. However, many stones are found in the fundal portion of the 2 chambers, and this suggests that motility is impaired in the fundal segment where bile stasis occurs.^19^ In fact, when those with bicameral GB (n = 8) were taken into account, only 25% (2/8) of patients showed response to dissolution therapy: hypodense stone, 0% (0/2); isodense stone, 33.3% (1/3); hyperdense stone, 50% (1/2); and calcified stone 0% (0/1). Those who showed response to dissolution therapy despite the presence of bicameral GB were those with narrowed segment of GB that was larger than that of the stone diameter. And even when there was response, none of them showed complete response but only partial response was observed.

In addition to isodense stones, sludge also showed high response and dissolution rates to dissolution therapy. Interestingly, however, 50% of calcified sludge showed partial response to dissolution therapy. Because calcified stones (or sludge) are known to be unresponsive to dissolution therapy, partial response shown in this study seems to have been due to the passage of stone/sludge rather than the occurrence of actual dissolution.

In the present study, response and dissolution rates between CNU and UDCA were also compared. The dissolution efficacy of UDCA alone or in combination with CDCA has been shown to be comparable in a meta-analysis.^12^ However, the majority of studies have been conducted with the Western population, and data on the efficacy of oral litholysis are lacking with the Eastern population, which was one of the reasons for carrying out this study. The recommended dose of UDCA for gallstone dissolution is 8 to 12 mg/kg/d,^7^ and the dose for the combination of UDCA and CDCA in previous studies was 5 mg/kg/d each or 6 mg/kg/d each.^7,20,21^ In the present study, 600 mg/dof UDCA was given, and 3 capsules of CNU (each capsule containing 114 mg of CDCA and 114 mg of UDCA) were prescribed for 6 months, as these are the dose of bile acids for gallstone dissolution that is covered by the National Health Insurance Program of Korea.^8^ If we assume that dissolution efficacy of equimolar dose of UDCA and CDCA to be similar, patients prescribed with CNU would have received 684 mg/d of bile acid whereas patients prescribed with UCDA received 600 mg/d of bile acid. Because the total dose of CNU was higher, the dissolution efficacy could have been expected to be better in the CNU group. However, UDCA performed slightly better than CNU, although not statistically different. This might be due to the fact that there were more patients with sludge in the UDCA group.

There are several limitations in the present study. First, the retrospective nature of this study limited the evaluation of some of the variables which could have influenced dissolution efficacy. Changes in gallstone-related symptoms also could not be assessed. Second, the duration of dissolution therapy was 6 months. If the study period had been longer, the response and dissolution rates could have increased, especially for isodense stones. Not being able to assess recurrence rate after dissolution therapy is also another limitation. Third, GB function was not evaluated. Uncomplicated gallstones are more commonly attended by primary care physicians, and measuring GB function by diisopropyl iminodiacetic acid scan is not always possible, and cholecystogram is an obsolete practice. However, there is no doubt that knowing GB function would help to select more appropriate candidates for gallstone dissolution.

## CONCLUSIONS

Isodense gallstones and sludge showed high response rate and dissolution rate to oral litholysis with UDCA and CNU showing comparable efficacy. Although it is not possible to know the exact composition of GB stone until it has been operated, visibility of gallstone on CT scan strongly suggests deposition of calcium and the chance of gallstone dissolution decreases. Although isodensity does not always imply the presence of cholesterol stone, the likelihood of GB stone being cholesterol stone could increase with the help of CT scan. Thus, CT scan is recommended before initiating oral litholysis if gallstone dissolution is intended.
